# Investigation of a novel *PROS1* splicing variant in a patient with protein S deficiency

**DOI:** 10.1038/s41439-024-00286-9

**Published:** 2024-07-26

**Authors:** Yo Niida, Wataru Fujita, Sumihito Togi, Hiroki Ura

**Affiliations:** 1 Center for Clinical Genomics, Kanazawa Medical University Hospital, Ishikawa, Uchinada, Japan; 2 Division of Genomic Medicine, Department of Advanced Medicine, Medical Research Institute, Kanazawa Medical University, Ishikawa, Uchinada, Japan; 3 Department of Cardiology, Kanazawa Medical University, Ishikawa, Uchinada, Japan

**Keywords:** Gene expression, Thromboembolism

## Abstract

Here, we report a novel *PROS1* splicing mutation in a patient with type I protein S deficiency. Qualitative and quantitative analysis of pathogenic splicing variants at the mRNA level was performed by long-range PCR-based targeted DNA and RNA sequencing. A base substitution in the exon 4 splicing donor site activates a potential splicing donor site in intron 4, resulting in an in-frame insertion of 48 bases (16 amino acids).

Protein S (PS) is a vitamin K-dependent coagulation regulator that acts as a cofactor for protein C (PC), promoting PC inactivation of factor V by approximately 10-fold. In plasma, approximately 60% of PS is bound to the C4b binding protein (C4BP) b-chain, while the remaining 40% is free; the free form is believed to be the active form^1^. PS deficiency increases the risk of thrombosis, and it may occur secondary to liver disease, nephrotic syndrome, or antiphospholipid antibody syndrome or may be congenital due to loss-of-function mutations in the *PROS1* gene. Congenital PS deficiency is usually an autosomal dominant trait (OMIM #612336), but an autosomal recessive form can occur rarely with more severe manifestations (OMIM #614514)^2^. Patients with this condition mainly have a greater propensity to develop deep vein thrombosis and subsequent pulmonary embolism, but arterial embolism, such as stroke, may sometimes occur^3^, and these severe embolisms may constitute the initial presentation of the disease. In female patients, it can also cause fetal death during pregnancy^4^. *PROS2P* is a *PROS1* pseudogene with 97% homology, which causes problems in genetic testing. *PROS2P* lacks exon 1 and is thought to be nontranscribed^5^. Although PS activity is always reduced in PS deficiency, it is classified into three types according to the amount of antigen: type I (both total and free levels are reduced), type II (both total and free levels are normal), and type III (total level is normal, and free level is reduced)^1^.

A 58-year-old man was referred to our emergency department complaining of progressive dyspnea for 1 week. He was 187 cm tall, weighed 132 kg, and was obese, with a body mass index (BMI) of 37.75. The patient’s oxygen saturation had decreased to 87%, and a chest X-ray revealed hyperlucency in the right upper lung. A pulmonary embolism was suspected. Although the patient had no history of thrombosis, his father had a history of pulmonary embolism (Fig. 1a). Contrast-enhanced CT revealed bilateral pulmonary artery thrombi and a thrombus extending from the inferior vena cava to both common iliac veins (Fig. 1b). On admission, complete blood count, liver function, and renal function were normal, but a blood coagulation test revealed increased levels of fibrin/fibrinogen degradation products (FDPs) and D-dimer (13.8 (≦4) and 9.60 (≦1.0) µg/ml, respectively). Anticoagulant therapy was started immediately, and an inferior vena cava filter was placed to prevent the progression of the pulmonary embolism. Subsequent tests revealed negative results for autoantibodies and normal activity of PC and antithrombin III. Specifically, PS activity (prothrombin time-based clotting method) decreased to 41% (67–146%), and free and total PS antigens (latex immunoassay) decreased to 37% (50–131%) and 48% (73–137%), respectively. These results were consistent with congenital PS deficiency type I. After the patient recovered, genetic counseling and *PROS1* genetic testing were performed to confirm the diagnosis.

**Fig. 1:**
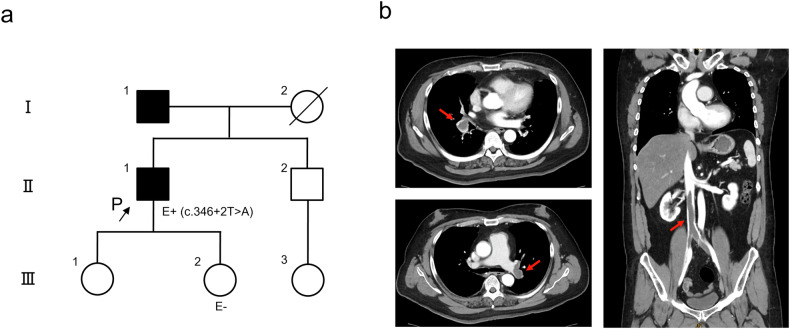
Pedigree and contrast-enhanced computed tomography of the patient. **a** Pedigree of the patient. P proband, E+ positive evaluation of the gene test, E− negative evaluation of the gene test. **b** Large thrombi were observed in the bilateral pulmonary arteries and inferior vena cava to the bilateral common iliac veins (red arrows).

To analyze the *PROS1* gene, combined long amplicon sequencing (CoLAS) was performed as previously reported^6,7^. CoLAS is a simultaneous targeted DNA and RNA sequencing strategy based on long-range PCR-based next-generation sequencing (NGS). Briefly, 20 ng of genomic DNA was amplified using five sets of long PCR primers (Supplementary Table 1) at a final concentration of 0.15 µM and KOD One DNA polymerase (TOYOBO, Osaka, Japan) to cover the *PROS1* genomic region, including all exons. Touchdown PCR cycles were performed under the following conditions: 3 cycles of 98 °C for 10 s and 74 °C for 10 min; 3 cycles of 98 °C for 10 s and 72 °C for 10 min; 3 cycles of 98 °C for 10 s and 70 °C for 10 min; and 25 cycles of 98 °C for 10 s and 68 °C for 10 min. Total RNA from peripheral blood mononuclear cells (PBMCs) was extracted with TRIzol (Thermo Fisher Scientific), and full-length double-stranded cDNA was synthesized from 50 ng of total RNA using a SMART-Seq® HT kit (Takara Bio USA, Mountain View, CA) according to the manufacturer’s standard protocol. Full-length PROS1 cDNA was amplified with long RT-PCR primers (Supplementary Table 1) at a final concentration of 0.15 µM and KOD One, using touchdown PCR cycles identical to those for DNA except for the 3-min extension time.

The NGS library was prepared from the long PCR products using an Illumina DNA prep with an enrichment kit (Illumina, San Diego, CA), and a 12.5 pM library was sequenced on an Illumina MiSeq system (2 × 250 cycles) following the standard Illumina protocol. Data analysis was performed as previously reported^7^. In brief, haplotype variant calling was performed using GATK’s HaplotypeCaller (version 4.0.6.0)^8^, RNA sequences were aligned with the reference human genome (hg38) using HISAT2 (version 2.1.0)^9^, and the Integrative Genomic Viewer (IGV version 2.4.13) was used for visualization^10^. Since the genomic orientation of PROS1 is opposite, the original IGV image shows the complementary strands. Figure 2 is intentionally inverted to facilitate understanding of splicing variants. The detected variant was validated by direct DNA sequencing using the *PROS1* exon 4-specific primer set (Supplementary Table 1) and the BigDye Terminator v3.1 cycle sequencing kit on the ABI PRISM 3100xl genetic analyzer (Thermo Fisher Scientific).

**Fig. 2:**
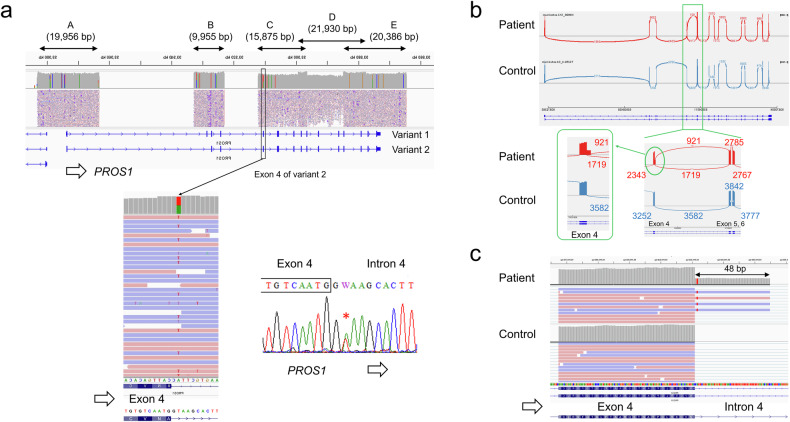
*PROS1* CoLAS analysis. **a** IGV image of DNA-level analysis. A base substitution of the intron 4 splice donor site, c.346+2 T > A, was detected and verified by Sanger sequencing. **b** IGV Sashimi plot. RNA level analysis revealed aberrant splicing in intron 4 resulting from the insertion of intron sequences into the PROS1 mRNA. Each number indicates the number of splice sites. **c** IGV image of RNA analysis showing in-frame insertion (48 bp). Note that all insertion reads are from the variant allele (c.346+2A). Note that the original IGV image of the NGS analysis shows complementary strands because the orientation of *PROS1* on the genome is reversed. In the figure, the IGV images have been reversed for ease of understanding. In **a**, **c**, the variant allele is shown as T (red), the complement of A.

DNA sequence analysis revealed a novel splicing variant of *PROS1*, NG_009813.1(NM_000313.4):c.346+2 T > A, in the patient (Fig. 2a). This variant is located at a consensus splice donor site and is predicted to affect *PROS1* mRNA splicing. RNA level analysis revealed that a 48-base insertion had occurred between exons 4 and 5, with GT at +49_ + 50 of intron 4 serving as a new splicing donor site. Approximately 98% of the *PROS1* mRNA expressed in blood was variant 2 (NM_000313.4), and almost no transcription of variant 1 (NM_001314077.2) was observed. At the protein level, this variant results in an in-frame insertion of 16 amino acids (GKHFYHQLKKQNKNSV) (Fig. 2b, c, Supplementary Fig. 1a, b). According to HGVS nomenclature, this variant can be written as NG_009813.1(NM_000313.4):c.346+2 T > A r.346_347ins[346 + 1_346 + 48] p.Asn115_Ala116insX[16]. Interestingly, according to the IGV Sashimi plot, the number of junction reads indicating aberrant splicing of the 42-base insertion (921) was not reduced (54%) as much as the number of junction reads for normal splicing of exons 4 to 5 (1719). It is suggested that much of the mRNA from the variant allele escapes nonsense-mediated RNA decay because the in-frame insertion has no stop codon^11^. Since this patient was type I and the amount of PS antigen was reduced to half, it is believed that a posttranslational effect may affect the decrease in protein level. In fact, there are several reports of decreased protein stability due to missense^12^ and in-frame^13^ variants. In addition, the protein level in this case was measured by immunoantigenicity; therefore, it is possible that the in-frame insertion of the 16 amino acids changes the antigenicity of the protein rather than the level. When the variant allele is expressed as a protein, the 16 amino acid insertion occurs just before the first epidermal growth factor-like domain of protein S (Supplementary Fig. 1c). In any case, PS activity is reduced, and the variant effect is deleterious. The effects of in-frame deletions/insertions of mRNA variants at the protein level previously reported in similar cases vary. Leroy-Matheron et al. reported an intron 5 variant, c.469+5 G > A, causing both an exon 5 skip (123 bp) and an exon 5 and 6 skip (255 bp); the expression of two truncated proteins was confirmed by immunoblot, and the patient exhibited type II PS deficiency^14^. Nagaya et al. reported that c.345+5 G > C caused aberrant splicing of a 48 bp insertion of the intron 4 sequence between exon 4 and exon 5. The patient exhibited type III PS deficiency because the total PS antigen level was normal, but the free PS antigen level was decreased^15^. On the other hand, Misukami et al. reported a type I patient with c.602-2 A > T causing an exon 7 skip (126 bp) and a decreased total PS antigen level^16^. In addition, Tan et al. reported that c.965+4 A > T causes a 78 bp deletion within exon 9 and a reduced expression level of deleted mRNA^17^.

The variant reported in our case is absent in the general Japanese population according to the Japanese Multi Omics Reference Panel (jMorp, https://jmorp.megabank.tohoku.ac.jp/) and the Genome Aggregation Database (gnomAD, https://gnomad.broadinstitute.org/). Additionally, this variant has not been registered in ClinVar (https://www.ncbi.nlm.nih.gov/clinvar/) and was registered by us for the first time (in progress as of November 12, 2023). This variant is classified as likely pathogenic according to the ACMG/AMP guidelines^18^ (PM1 [insertion is located between the thrombin sensitive region (TSR) domain and the first epidermal growth factor-like (EGF) domain] ^1^ + PM2 [absent from controls] + PM4 [protein length changes as a result of in-frame insertions in a nonrepeat region] + PP3 [multiple lines of computational evidence support a deleterious effect] + PP4 [phenotype is highly specific for a disease]). After the pathogenic variant was confirmed, the patient’s second daughter requested genetic counseling and testing, and the test results were negative (Fig. 1a). The father had no intention to undergo genetic testing.

Currently, capture probe methods are often used for genetic testing by NGS, but long-range PCR-based NGS has several advantages. As in this case, when a pseudogene (*PROS2P*) is present, it is difficult to distinguish it from the true gene (*PROS1*) using the capture probe method, but with long-range PCR-based NGS, it is possible to exclude *PROS2P* by designing PCR probe sets that are highly specific for *PROS1*. The specificity of the primers used in this study was verified using Primer Blast (https://www.ncbi.nlm.nih.gov/tools/primer-blast/, last accessed November 10, 2023). Removing the need to capture probes saves time and eliminates off-target reads. Furthermore, by amplifying full-length cDNA with long-range reverse transcribed PCR and performing NGS analysis, it is possible to measure the effects of splicing abnormalities across the entire target gene qualitatively and quantitatively. This study shows that the base substitution of the splice donor site at position +2 activates a potential donor site within the intron, causing an in-frame insertion of 48 bases, and that this occurs at the same level as normal splicing. In this way, CoLAS allows efficient analysis of the consequences of splicing abnormalities. Because PROS1 gene expression occurs primarily in the liver, the use of PBMCs is a limitation of this study. However, the advantage of being able to directly observe which splicing abnormalities occur in the patient’s own biological tissues is significant. This method is applicable even when a single variant at the DNA level results in multiple abnormal mRNA variants^19^. The effects of splicing variants on mRNAs can be easily analyzed qualitatively and quantitatively using CoLAS.

## HGV Database

The relevant data from this Data Report are hosted at the Human Genome Variation Database at 10.6084/m9.figshare.hgv.3415.

## Supplementary information

Supplementary Figure 1. The outcome of the splicing variant of the present case.

Supplementary Table 1. PROS1 PCR Primers

## Data Availability

The *PROS1* splicing variant reported in this study was registered in ClinVar (https://www.ncbi.nlm.nih.gov/clinvar/, last accessed November 10, 2023), with the submission ID SCV004101027.
