# Supplementary material for: Investigation of a novel PROS1 splicing variant in a patient with protein S deficiency
Source: Hum Genome Var. 2024 Jul 26;11:28. doi: 10.1038/s41439-024-00286-9 (PMC11282053; doi:10.1038/s41439-024-00286-9)
Supplement: Supplementary file 1 — Supplementary Figure 1. The outcome of the splicing variant of the present case. [file 41439_2024_286_MOESM1_ESM.pdf]

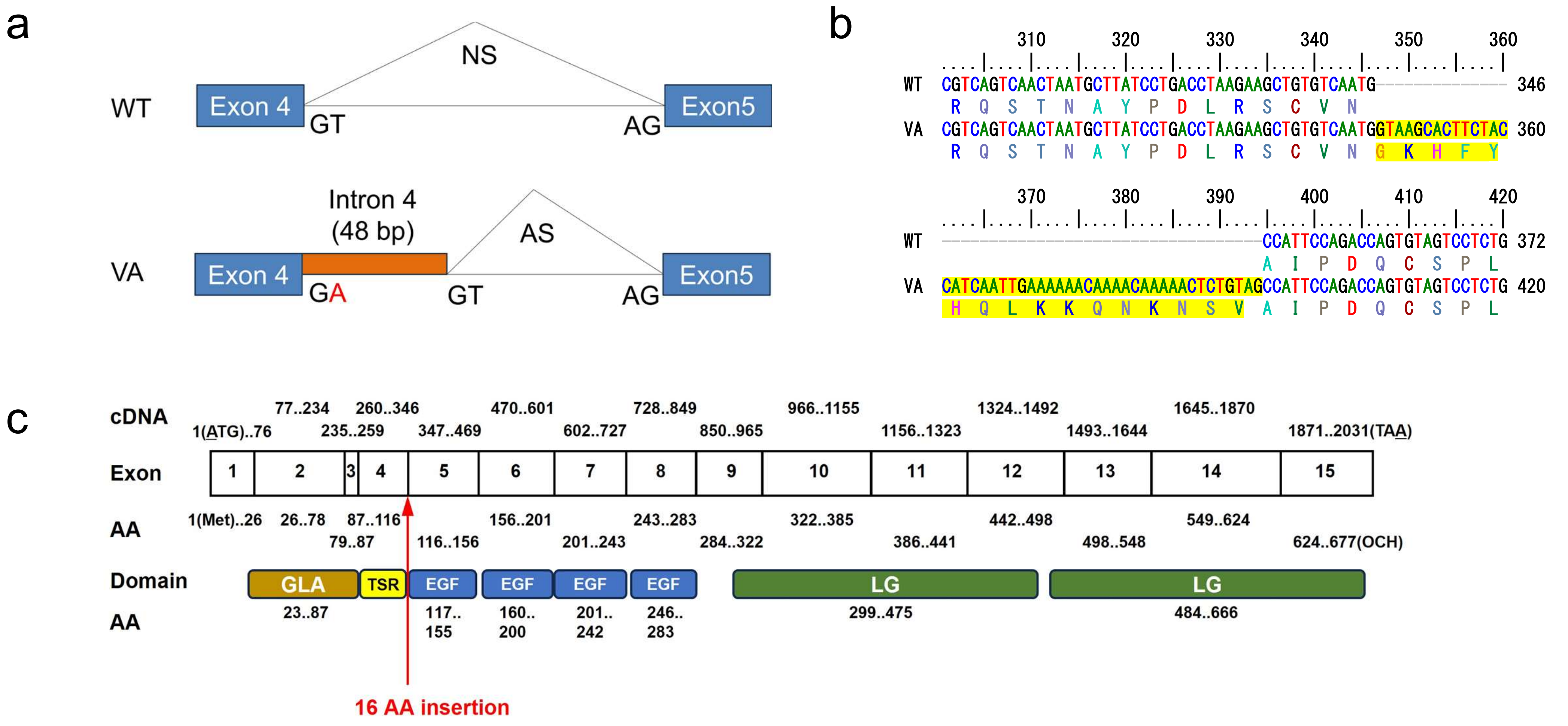

Supplementary Figure 1. The outcome of the splicing variant of the present case. (a) Schematic representation of the aberrant splicing of PROS1. The GT to GA change of the intron 4 splice donor site activates a criptic donor site in intron 4, NG_009813.1(NM_000313.4):c.346+49_346+50GT, causing a 48 bp insertion of the intron 4 sequence into the mRNA. (b) Translation of a 48 bp insertion. The aberrant 16 amino acids are inserted between Asn (N) 115 and Ala (A) 116. (c) Schematic representation of the cDNA, amino acids, exon number and protein domain positions. The aberrant insertion of 16 amino acids is located just before the first EGF-like domain. AA; amino acid, AS; aberrant splicing, EGF; epidermal growth factor-like domain, GLA; gamma-carboxyglutamic acid-rich domain, LG; laminin G domain, NS; normal splicing, TSR; thrombin sensitive region domain, WT; wild type, VA; variant allele.
