# Supplementary material for: Investigation of a novel PROS1 splicing variant in a patient with protein S deficiency
Source: Hum Genome Var. 2024 Jul 26;11:28. doi: 10.1038/s41439-024-00286-9 (PMC11282053; doi:10.1038/s41439-024-00286-9)
Supplement: Supplementary file 2 — Supplementary Table 1. PROS1 PCR Primers [file 41439_2024_286_MOESM2_ESM.pdf]

Supplementary Table 1. *PROS1* PCR Primers

Long-range PCR primers for DNA

Primer set	Primer Name	Primer sequence	Primer position (NC_000003.12 Homo sapiens chromosome 3, GRCh38.p14)	Product size (bp)
PROS1_DNA_Long_A	PROS1_L_A-F	5'-TCAGTACTCCACTAAGGGGGATGAAGC-3'	93983348_93983322	19,956
	PROS1_L_A-R	5'-CACAGGTTGGTCACTCTCTTGACTTCC-3'	93963393_93963419	
PROS1_DNA_Long_B	PROS1_L_B-F	5'-AGAGTTGGGCAGGGCCAGAAAAGC-3'	93923177_93923200	9,955
	PROS1_L_B-R	5'-GGCCAACCCAAGTGCTAGCCAAAGA-3'	93933131_93933107	
PROS1_DNA_Long_C	PROS1_L_C-F	5'-CCCCTGCAAACTGCTCCGCCA-3'	93896616_93896636	15,875
	PROS1_L_C-R	5'-AATGACCTCCTTCCTCCACCCTGCC-3'	93912490_93912466	
PROS1_DNA_Long_D	PROS1_L_D-F	5'-AACCAGGGATTGCCACCAGCACG-3'	93877690_93877712	21,930
	PROS1_L_D-R	5'-CAGCCCCCGCAACACATTTCTCCTT-3'	93899619_93899595	
PROS1_DNA_Long_E	PROS1_L_E-F	5'-TGAGGGCTTGTGATAGTATGAGCTTGC-3'	93885106_93885080	20,386
	PROS1_L_E-R	5'-CAGAGCTCACTGTAGCAGACATGAACG-3'	93864721_93864747	

Long-range PCR primers for RNA

Primer set	Primer Name	Primer sequence	Primer position (XM_054347264.1 Homo sapiens protein S (PROS1), transcript variant X1, mRNA)	Product size (bp)
PROS1_RNA_Long	PROS1_LR-F	5'-GTCTCAGCAGTGTTTACTAGGC-3'	499_520	2,862
	PROS1_LR-R	5'-CCATCTCTTCTGCCTTCATCAG-3'	3360_3339	

Exon 4 specific primers

Primer set	Primer Name	Primer sequence	Primer position (NC_000003.12 Homo sapiens chromosome 3, GRCh38.p14)	Product size (bp)
PROS1_Ex4_specific	PROS1_Ex4_F	5'-TACCTCTTGGGACAGTTCCTAC-3'	93910789_93910768	310
	PROS1_Ex4_R	5'-TGAAAATACTTCCTTGCTGGGC-3'	93910480_93910501	
